# Signet-Ring Cell Morphotype in Lung Carcinoma

**DOI:** 10.5146/tjpath.2019.01475

**Published:** 2020-09-15

**Authors:** Adriana Handra-Luca

**Affiliations:** APHP GHU Avicenne, Bobigny, France

**Dear Editor,**

**Figure 1:**
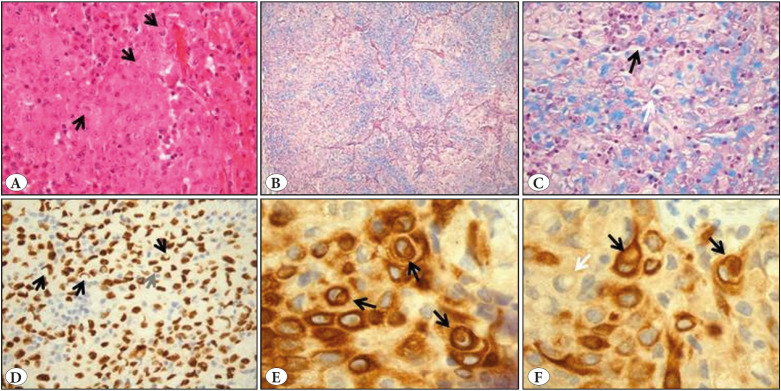
**A)** The lung tumor was of non-small cell carcinoma type showing a solid pattern (black arrow for cytoplasmic vacuole) (H&E; x40). **B,C)** The Alcian blue stain showed numerous, positive vacuoles (black arrow). The presence of positive vacuoles in perinuclear location was noted (white arrow) (Alcian Blue; x10 & x40). **D)** TTF1 was expressed by the «compressed nuclei» (black arrows) with intranuclear inclusions (gray arrow). (IHC ; x40). **E,F)** Cytokeratin 5/6 was expressed in intracytoplasmic vacuoles of the tumor cells of signet-ring cell morphology (black arrows). Cytokeratin 5/6-negative signet-ring-cells were admixed with the CK5/6-positive cells (white arrow) (IHC; x1000).

We have read with great interest the article by Yigit et al. on the signet-ring cell morphotype in lung TTF1-negative p63-positive squamous cell carcinoma (1). Cytokeratin (CK) 5/6 was not expressed in the cytoplasmic vacuoles. We recently had the opportunity to detect CK5/6 in signet-ring cells in a non-small cell lung carcinoma of mixed subtype (with extensive solid pattern) (Figure 1). The tumor showed mucus secretion. The tumor cells expressed TTF1 and CK7 diffusely. p63 was expressed in sparse cells. CK5/6 was expressed in several tumor foci, in agreement with the CK5/6 immunoheterogeneity reported by Rekhtman et al. (2). CK5/6-positive signet-ring cells were admixed with CK5/6-negative signet-ring cells. Alcian blue staining was positive for the signet-ring-type cells. The presence of CK5/6 in intracytoplasmic vacuoles of similar shape and size to those detected on the hematoxylin-and-eosin and Alcian-blue stains is difficult to explain: both CK5 and CK6 are basic, type II keratins while the Alcian blue staining detects acidic polysaccharides. Since the immunohistochemistry and special stainings were not performed on the same tissue section, an expression of CK5/6 in an Alcian blue-positive intracytoplasmic vacuole is difficult to prove. Moreover, the signet-ring cell appearance, consisting of a large cytoplasmic vacuole compressing the nucleus, could be rather the result of cytoskeletal changes, independent from the substance composing the cytoplasmic vacuole acidic polysaccharides of mucins, polypeptides of keratin-type, or the lipids of adipocytes. Cytoskeletal reorganisation similar to that reported for vimentin during adipose conversion could be one explanation (3).
